# Supplementary material for: Learning curve and surgical outcome of robotic assisted colorectal surgery with ERAS program
Source: Sci Rep. 2022 Nov 29;12:20566. doi: 10.1038/s41598-022-24665-w (PMC9709162; doi:10.1038/s41598-022-24665-w)
Supplement: Supplementary file 2 — Supplementary Information 2. [file 41598_2022_24665_MOESM2_ESM.zip › RAL Raw data-Ñ[▒K/107/OP N_ñw╝╨░O▒Kñσ.pdf]

W52 / W52

臺中榮民總醫院
手術室護理記錄

[redacted]

基本資料

- 室別： 開刀房-17室，手術科別： CRS，手術類別： 常規，日期： 2021/08/20
- 進入時間： 0720 ； 麻醉開始時間： 0756 ； Time Out時間： 0900 ； 劃刀時間： 0901 ； 手術結束時間： 1340 送出時間： 1350 ；
- 麻醉方式： GE
  診斷：rectum ca 10 cm FAV
  手術名稱：Robot Assisted Colectomy(L)
  手術局部用藥：nil
  術前預防性抗生素：
  - 抗生素名稱一： Veterin 1GM IVD/由麻醉護理人員給予
  - 時間： 0840
  - 上次簽章: 歐真勤

  ，追加預防性抗生素：
- 手術醫師： 林俊余，陳映菁，郭連雄，陳瑋夫
- 麻醉醫師： VS 陳永銘
-
  - 刷手護士A ： 吳婉寧，時間： 0720，刷手護士B ： 陳盈妤，時間： 0720，接替刷手一： 張秀敏，時間： 1200，接替刷手二： 童歐文，時間： 1200
  - 流動護士A ： 歐真勤，時間： 0720，接替流動一： 盧陵怡，時間： 1200

皮膚狀況

A-V Shunt： 無
術前： 完整
記錄者： 上次簽章: 歐真勤
術後： 完整
記錄者： 盧陵怡

手術準備

手術臥式 單一手術臥式

-
  - 手術臥式： 截石臥式
  - 使用附件： 溫毯 38 ℃，頭圈，腳架
  - 皮膚準備： 10%Aq Betadine，Hibiscrub，2% Chlorhexidine
  - 電燒： 使用電燒: 電極片位置： 其它： 導電軟墊，雙極電燒，記錄者： 上次簽章: 歐真勤

止血帶T： 無

植入物

無

標本送檢

- 細菌培養 無
- 細胞學 無
- 冰凍切片 無
- 病理標本
  組織名稱一：rectum
  ,
  組織名稱二：IMA root
  ,
  組織名稱三：distal cut-end
- 截肢 無

標本交付 標本： 3 袋，標本： 0 盒，標本： 0 瓶，細胞學： 0 管，Culture Tube： 0 管，被交付醫師： VS 林俊余，

- 記錄者： 盧陵怡
- 時間： 1350

傷口術後數料

傷口數料： 傷口數料： 無

- 請於圖上標示部位：

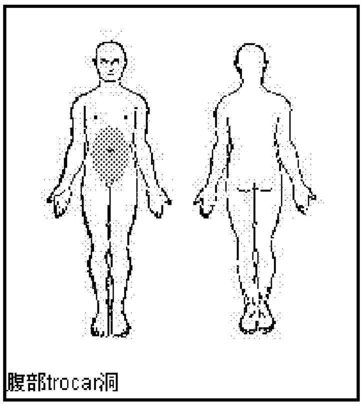

- 傷口數料： mepore

導管

導管	天數	導管資訊	插入日期	插入時間	更換日期	拔除日期	拔除時間	拔除原因	預計拔除/更換	預計拔除/更換日期
CVC(1)	1	號碼:8 Fr; 深度:15cm; 部位:jugular (右); 象限:RU;	2021/08/20	0820						
A-line (1)	1	號碼:20 Ga; 部位:左手;	2021/08/20	0800						
Epidural PCA(1)	1	部位:硬膜外;	2021/08/20	0810						
Sump NG Tube(1)	1	號碼:16 Fr.; 深度:65cm; 部位:胃;	2021/08/20	0815						
Hemo Vac(1)	1	號碼:15 Fr.; 部位:骨盆腔;	2021/08/20	1145						
Foley(1)	9	號碼:16 Fr.; 部位:尿道;	2021/08/12	0533						

用物計數

- 手術中出血量： MINIMAL c.c.
- 手術中尿量： 100 c.c.
-
  - 其它： 無

用物計數： 多切口同時縫合

- 1.器械： 正確，2.縫針： 正確，3.刀片： 正確，4.紗布： 正確，5.腹部墊： 未使用，6.紗球： 未使用，7.PEANUTS ： 未使用，8.紗條： 正確，9.絲帶： 未使用，10.針頭： 未使用，11.Vessel loop： 未使用，12.Boots： 未使用，13.Jelco： 未使用，14.棉條： 未使用，
  - 第一次：
    - 刷手護士 ： 吳婉寧
    - 流動護士： 上次簽章: 歐真勤
    - 時間： 1130

  ,
  - 第二次：
    - 刷手護士 ： 吳婉寧
    - 流動護士： 上次簽章: 歐真勤
    - 時間： 1150

  ,
  - 第三次：
    - 刷手護士 ： 童歐文
    - 流動護士： 盧陵怡
    - 時間： 1335

備註

備註： davinci0度scopy:NO1

- 記錄者： 上次簽章: 歐真勤
- 時間： 0900

填寫護理人員 盧陵怡

正式摘要

W52 / W52

doctorRank:B
